# Shear bond strength of a RMGIC for orthodontic bracket bonding to enamel

**DOI:** 10.1038/s41405-023-00181-5

**Published:** 2024-01-02

**Authors:** Maureen Boudrot, Philippe François, Sarah Abdel-Gawad, Jean-Pierre Attal, Claire-Adeline Dantagnan

**Affiliations:** 1 Pitié Salpêtrière Hospital, 47-83 Boulevard de l’Hôpital, 75013 Paris, France; 2 Innovative Dental Materials and Interfaces Research Unit (URB2i), UR 4462, 1 Rue Maurice Arnoux, 92120 Montrouge, France; 3 Bretonneau Hospital, 23 Rue Joseph de Maistre, 75018 Paris, France; 4 Charles Foix Hospital, 7 Avenue de la République, 94200 Ivry-sur-Seine, France

**Keywords:** Dental materials, Orthodontics

## Abstract

**Objective:**

To evaluate the shear bond strength (SBS) of a restorative resin-modified glass ionomer cement (RMGIC) for orthodontic bracket bonding.

**Materials and methods:**

One hundred twenty-one human teeth were randomly divided into 11 groups (*n* = 11) according to the surface treatment applied (H_3_PO_4_ ± Transbond Plus (TSEP) or Scotchbond Universal (SU)), and the adhesive used (Riva LC HV (RIVA), Fuji Ortho (FUJI), and Transbond XT (TXT)). For each sample, a metal button was bonded. SBS tests were performed at 1 week and debonded specimens were observed for failure modes determination. One-way ANOVA followed by Tukey’s post hoc test was used to compare SBS differences and Fisher’s exact test to analyze the failure modes (*p* < 0.05).

**Results:**

TSEP + FUJI and H_3_PO_4_ + SU + TXT showed the highest SBS values while H_3_PO_4_ + TSEP + RIVA showed the lowest value. Cohesive failure and mixed failure were found in the groups with SU and TXT and adhesive failure in the other groups.

**Discussion/Conclusions:**

The bonding of orthodontic attachments to enamel could be performed with any of the three materials studied. The use of a universal adhesive in the bonding protocol could optimize the adhesion values. Clinical studies would be needed to confirm the results obtained.

## Introduction

In orthodontics, bonding materials must have general properties due to their use in oral cavity (biocompatibility, resistance to solubility and to chemical or physical–chemical attack by the oral environment) [1, 2] and specific properties related to their application (sufficient retention to withstand masticatory forces and those delivered by the appliance, immediate watertightness, sufficient working time, easy removal of excess, tolerance to handling and easy debonding without damaging the enamel surface) [1, 2]. Reynolds [3] estimated that the minimum adhesion values needed in orthodontics reached 5.9 to 7.8 MPa, but this value had to be weighed against the fact that these results varied from one experimental device to another [4].

To date, three main families of bonding materials are used in orthodontics: composite resins combined with different primer systems, resin-modified glass ionomer cements (RMGICs) and self-adhesive cements [1, 2]. Among the available orthodontic bonding materials, composite resins are the most frequently employed [1, 2]. These materials are easy to handle and provide adequate bonding values with good clinical results, as demonstrated by many studies [1, 2, 5]. However, there are disadvantages to their use, including the absence of fluoride release with the risk of white spot lesions appearing around the brackets at the end of the treatment [6] and bonding difficulties in cases of salivary and blood contamination [7, 8].

To overcome the disadvantages of composite resins, the use of glass ionomer cements (GICs) has been proposed because of their ability to chemically bond to the hydroxyapatite of the enamel and to release fluoride, which is useful for preventing white spot lesions around brackets [9]. However, the first generations of conventional GICs show unacceptable debonding rates compared with composite resins [10–13]. For this reason, the development of these materials has led to the introduction of RMGICs in orthodontics. These materials, with their improved mechanical properties, offer several advantages compared with composite resins [14–17]: prevention of white spot lesions [18] by fluoride release [19] and cariogenic bacterial growth inhibition [20], reduction in the risk of iatrogenic damage to the enamel during debonding [20, 21], preservation of weakened enamel (molar incisor hypomineralization, amelogenesis imperfecta, etc.) [22] and tolerance to humidity [20, 21].

However, although many authors support the use of RMGICs for bonding orthodontic brackets, the data in the literature remain controversial, with clinical studies reporting more frequent debonding than with the use of a primer/composite resin combination [23–25].

The aims of this in vitro study are to assess whether Riva LC HV (SDI), which is a restorative RMGIC, can be a competitor to either Fuji Ortho LC or the adhesive/Transbond XT combination (3 M Unitek) for bonding orthodontic attachments to enamel and to determine which bonding protocol(s) can optimize their adhesion values.

## Materials and methods

### Materials tested

Three materials—one orthodontic composite resin and two RMGICs (one restorative and one orthodontic)—were studied and compared:

- Transbond XT (3M Unitek).
- Fuji Ortho LC (GC Corporation).
- Riva LC HV (SDI).

These materials were combined with different adhesive systems (a one-step self-etching adhesive and a universal adhesive):

- Transbond Plus (3M Unitek).
- Scotchbond Universal (3M ESPE).

All tested materials and their manufacturers and chemical compositions are listed in Table 1.

**Table 1:** Materials, manufacturers and chemical compositions.

Materials	Type	Name	Abbreviation	Manufacturer	Composition
Primers	One-step SEP	Transbond Plus	TSEP	3M Unitek, Saint Paul, MN, USA	Silane-treated glasses: 65–75% Citric acid dimethacrylate oligomer: 5–10% 1–3 Glycerol DMA: 10–20% Silica: <2% Diphenyliodonium hexafluorophosphate: <0.5%
	Universal adhesive	Scotchbond Universal	SU	3M ESPE, Saint Paul, MN, USA	10-MDP: 1–10% PAC: 1–5% HEMA: 15–25% DMAEMA: 5–15% Bis-GMA: 15–25% EDMAB Ethanol: 10–15% Water: 10–15% Camphoroquinone Silane: 5–10% Fillers
		Fuji Ortho Conditioner	OC	GC Corporation, Tokyo, Japan	Polyacrylic acid: 10%
Adhesives	Composite resin	Transbond XT	TXT	3M Unitek, Saint Paul, MN, USA	Bis-GMA: 45–55% TEGDMA: 45–55% 4-(Dimethylamino)-Benzeneethanol: <0.5%
	RMGIC	Fuji Ortho LC	FUJI	GC Corporation, Tokyo, Japan	Polyacrylic acid: 5–10% Tartaric acid: 1–2.5% Fluoroaluminosilicate: 100% HEMA: 5–10% UDMA: 1–2.5% 2-Hydroxy-1,3 dimethacryloxypropane: 1–2.5% Initiators
	RMGIC	Riva LC HV	RIVA	SDI, Bayswater, VIC, Australia	Polyacrylic acid: 15–25% Tartaric acid: 1–5% HEMA: 20–25% Dimethacrylate crosslinker: 10–25% Acid monomer: 10–20% Fluoroaluminosilicate: 95–100%

### Sample preparation and bonding protocols

One hundred and twenty-one freshly extracted human molars and premolars were collected, cleaned of residual soft tissues, stored at 4 °C in a 1% chloramine T solution before usage and used within 3 months. Teeth were obtained from the dental departments of AP-HP, France. All experiments were conducted in accordance with the principles articulated in the Declaration of Helsinki. All teeth were collected with the informed oral consent of all patients, which was in accordance with the ethical guidelines set by French law and with specific authorization by a university dental school in Paris (no. DC- 2009-927, Bioethic cell, DGRI/A5, Paris, France). The selection criteria for the teeth were the absence of cracks, restorations, and caries.

First, for tooth preparation, most of the roots were removed using a polisher with water-cooled sandpaper (80 grit). Then, the vestibular or lingual surfaces of the crowns were abraded in the same manner (800-grit sandpaper) to obtain a flat enamel surface (>7 mm^2^). The prepared teeth were placed in cylindrical plastic molds (25-mm diameter and 15-mm height) and covered with a self-curing acrylic resin (Plexil-Escil, Chassieu, France) to expose the flattened enamel surface. Prior to the bonding procedure, each sample was observed under an optical microscope (×40 magnification) to ensure that the exposed surface was free from debris.

Each sample was randomly divided into 11 groups (*n* = 11) according to the different bonding protocols applied, as summarized in Table 2.

**Table 2:** Presentation of the different bonding protocols applied for each group.

Groups	Bonding Protocols
Group 1 (G1, FUJI)	**Fuji Ortho LC**^a^ + 10 s light curing (Radii Expert lamp, SDI)
Group 2 (G2, OC + FUJI)	**Fuji Ortho Conditioner** 20 s + Rinsing/Drying 15 s + **Fuji Ortho LC**^a^ + 10 s light curing
Group 3 (G3, TSEP + FUJI)	**Transbond Plus** 3–5 s + Gentle drying 1–2 s + **Fuji Ortho LC**^a^ + 10 s light curing
Group 4 (G4, TSEP + PHOTO + RIVA)	**Transbond Plus** 3–5 s + Gentle drying 1–2 s + 10 s light curing + **Riva LC HV**^a^ + 10 s light curing
Group 5 (G5, TSEP + RIVA)	**Transbond Plus** 3–5 s + Gentle drying 1–2 s + **Riva LC HV**^a^ + 10 s light curing
Group 6 (G6, TSEP + TXT)	**Transbond Plus** 3–5 s + Gentle drying 1–2 s + 10 s light curing + **Transbond XT** + 10 s light curing
Group 7 (G7, H_3_PO_4_ + TSEP + RIVA)	**H**_**3**_**PO**_**4**_ **37% etching** 30 s + Rinsing/Drying 15 s + **Transbond Plus** 3–5 s + Gentle drying 1–2 s + 10 s light curing + **Riva LC HV**^a^ + 10 s light curing
Group 8 (G8, SU + TXT)	**Scotchbond Universal** 20 s + Gentle drying 1–2 s + 10 s light curing + **Transbond XT** + 10 s light curing
Group 9 (G9, H_3_PO_4_ + SU + TXT)	**H**_**3**_**PO**_**4**_ **37% etching** 30 s + Rinsing/Drying 15 s + **Scotchbond Universal** 20 s + Gentle drying 1–2 s + 10 s light curing + **Transbond XT** + 10 s light curing
Group 10 (G10, H_3_PO_4_ + SU + RIVA)	**H**_**3**_**PO**_**4**_ **37% etching** 30 s + Rinsing/Drying 15 s + **Scotchbond Universal** 20 s + Gentle drying 1–2 s + 10 s light curing + **Riva LC HV**^a^ + 10 s light curing
Group 11 (G11 H_3_PO_4_ + RIVA)	**H**_**3**_**PO**_**4**_ **37% etching** 30 s + Rinsing/Drying 15 s + **Riva LC HV**^a^ + 10 s light curing

^a^For Fuji Ortho LC and Riva LC HV, the capsules were mixed 10 s with the vibrator (Rotomix, 3M ESPE, St Paul, MN, USA) according to manufacturers’ recommendations and then injected on the base of orthodontic buttons.

On each sample, a 7-mm^2^ cylindrical metal orthodontic button (Orthopartner, Montreuil, France) was bonded as described in bonding protocols (Table 2) and following manufacturers’ instruction for use of each material. After bonding the button, excess material was gently removed with a scalpel. All samples were stored in distilled water at 37 °C for 7 days before testing.

### Shear bond strength test

Shear bond strength (SBS) was determined using a universal testing machine (LRX, Lloyd Instruments, Fareham, UK). Shear force was applied to the button/enamel surface interface using a shear device employing a force parallel to the adhesive interface with a crosshead speed set at 0.5 mm min^−1^.

The SBS (in MPa) was then calculated by dividing the maximum force in newtons (N) by the surface area (S) of the metal button (7 mm^2^).

### Failure mode determination

The debonded specimens were observed under a binocular microscope (BZH10 Olympus, Hamburg, Germany) at ×30 magnification to determine the failure modes obtained and to classify them according to the following four types:

- CF-E: cohesive failure within the enamel.
- AF: adhesive failure at the interface between the bonding material and the enamel.
- MF: mixed failure (combined adhesive and cohesive fractures within the enamel).
- CF-B: cohesive failure within the orthodontic button.

### Three-point bending test for elastic modulus evaluation

Thirty bars (25 × 2 × 2 mm) of the three materials tested (*n* = 10) were made by using a 2 × 2 × 25 mm silicon mold (EXA’lence, GC Corporation) according to ISO 4049. The curing was performed with a LED curing light (Radii Expert lamp, SDI) through a transparent film applied on each mold. Curing time was set at 90 s in 3 times: 30 s at the top, 30 s in the middle and 30 s at the down for each bar. Material bars were then stored in distilled water for 2 weeks at 37 °C before performing the test to ensure that the maturation process, which required several hours for the GIC family, was mostly completed.

A 3-point bending test was carried out at ambient temperature using a universal testing machine (AGS-X, SHIMADZU, France). Each bar was placed in the center of a specific specimen holder, and the force was applied by the device (50-kN force cell) with a crosshead speed set at 1 mm min^−1^ until fracture occurred. For each sample, the flexural modulus (Ef) in GPa was calculated using the following formula: Ef = FL^3^/4BH^3^, where F is the maximum force in newtons (N), L is the distance between the sample holder supports, B is the width of each sample, and H is the height of each sample.

### Degree of conversion measurement

For each of the three materials analyzed, cylindrical samples (6 mm in diameter and 2 mm in height) were prepared using a suitable silicone mold. A total of 3 samples of each RMGIC studied were light-cured for 20 s (Radii Expert lamp, SDI) immediately after mixing the capsules, while 3 samples of each were evaluated in self-curing mode without initial light curing. The composite resin tested was analyzed only after light curing (*n* = 3). All samples were stored in distilled water at 37 °C protected from light for 1 week before measuring their degree of conversion (DC).

DC was measured by Fourier transform infrared spectroscopy (FTIR) with a NicoletTM iS10 spectrometer (Thermo Fisher Scientific, Waltham, MA, USA) in attenuated total reflectance (ATR) mode. Spectra were collected using OMNIC software (Thermo Electron Corporation, Waltham, MA, USA). After the background, all measurements were obtained in the spectral region from 500 to 4000 cm^−1^ with a resolution of 8 cm^−1^ and 64 scans. For each sample, the spectra were collected 3 times, and the DC was measured by the same operator to eliminate interoperator variability. The spectra of the three materials analyzed in the unpolymerized state were collected for reference.

For each spectrum, the absorbance peak heights of the C=0 ester double bonds (1720 cm^−1^) and the aliphatic C=C double bonds (1638 cm^−1^) were measured first in the unpolymerized state and then 1 week after polymerization. The DC was then determined by evaluating the changes in absorbance peak height ratios of aliphatic C=C bonds and C=O ester bonds according to the following formula:

$${{{{{\rm{DC}}}}}}=\left(1-\frac{{{{{{\rm{Abs}}}}}}\frac{{{{{{\rm{C}}}}}}={{{{{\rm{C}}}}}}\,1638\,{{{{{{\rm{cm}}}}}}}^{-1}\left({{{{{\rm{P}}}}}}\right)}{{{{{{\rm{C}}}}}}={{{{{\rm{C}}}}}}\,1720\,{{{{{\rm{c}}}}}}{{{{{{\rm{m}}}}}}}^{-1}\left({{{{{\rm{P}}}}}}\right)}}{{{{{{\rm{Abs}}}}}}\frac{{{{{{\rm{C}}}}}}={{{{{\rm{C}}}}}}\,1638\,{{{{{{\rm{cm}}}}}}}^{-1}\left({{{{{\rm{NP}}}}}}\right)}{{{{{{\rm{C}}}}}}={{{{{\rm{C}}}}}}\,1720\,{{{{{{\rm{cm}}}}}}}^{-1}\left({{{{{\rm{NP}}}}}}\right)}}\right)\begin{array}{c}{{{{{\rm{Abs}}}}}}:\,{{{{{\rm{absorbance}}}}}}\\ {{{{{\rm{P}}}}}}:\,{{{{{\rm{polymerized}}}}}}\\ {{{{{\rm{NP}}}}}}:\,{{{{{\rm{nonpolymerized}}}}}}\end{array}$$

### Statistical analysis

The results of the shear bond strength (SBS), flexural modulus (Ef) and degree of conversion (DC) tests were expressed by means and standard deviations, and they were summarized in an Excel table. The normal distributions of the variables were confirmed by the Shapiro‒Wilk test, and the equality of variances was verified by the Levene test.

Three one-way ANOVAs, followed by a Tukey post hoc test, were performed to assess the differences in SBS, Ef and DC values between the different groups or materials studied. Fisher’s exact testing for simple comparisons between groups and pairwise analysis were carried out for failure mode analysis.

All statistical tests were performed using XLSTAT software (Addinsoft, Paris, France). A significance level of *p* < 0.05 was set for all tests.

## Results

### Shear bond strength values

The SBS values obtained for all the groups are summarized in Table 3.

**Table 3:** Means and standard deviations of shear bond strength (SBS) obtained for each group according to the bonding protocol applied.

Groups	SBS values (MPa)
G1 (FUJI)	21.87 ± 5.6^cd^
G2 (OC + FUJI)	27.32 ± 7.38^bc^
G3 (TSEP + FUJI)	37.23 ± 3.84^a^
G4 (TSEP + PHOTO + RIVA)	22.03 ± 6.43^cd^
G5 (TSEP + RIVA)	17.54 ± 5.33^de^
G6 (TSEP + TXT)	19.38 ± 8.92^de^
G7 (H_3_PO_4_ + TSEP + RIVA)	17.51 ± 5.51^de^
G8 (SU + TXT)	21.6 ± 9.31^cd^
G9 (H_3_PO_4_ + SU + TXT)	29.30 ± 7.54^bc^
G10 (H_3_PO_4_ + SU + RIVA)	25.15 ± 3.61^bc^
G11 (H_3_PO_4_ + RIVA)	22.09 ± 5.34^cd^

Values with the same superscripted letter are not significantly different at *p* < 0.05.

Group 3 (TSEP + FUJI) had the highest SBS value (37.23 MPa *p* < 0.05), while Group 7 (H_3_PO_4_ + TSEP + RIVA) had the lowest SBS value (17.51 MPa *p* < 0.05). Group 2 (OC + FUJI) obtained a significantly higher SBS value than Group 6 (TSEP + TXT) (27.32 MPa vs. 19.38 MPa, respectively).

Among the Riva LC groups, the highest SBS value was obtained with Group 10 (H_3_PO_4_ + SU + RIVA) (25.15 MPa, *p* < 0.05), followed by Group 11 (H_3_PO_4_ + RIVA) (22.09 MPa, *p* < 0.05) and Group 4 (TSEP + PHOTO + RIVA) (22.03 MPa, *p* < 0.05), and the lowest SBS value was obtained with Group 7 (H_3_PO_4_ + TSEP + RIVA) (17.51 MPa *p* < 0.05), followed by Group 5 (TSEP + RIVA) (17.54 MPa, *p* < 0.05).

### Failure mode analysis

The failure modes obtained for all the groups are summarized in Table 4.

**Table 4:** Failure mode values obtained for each group.

Groups	AF	CF-E	MF	CF-B
G1 (FUJI)^b^	11	0	0	0
G2 (OC + FUJI)^b^	11	0	0	0
G3 (TSEP + FUJI)^b^	11	0	0	0
G4 (TSEP + PHOTO + RIVA)^ab^	9	0	2	0
G5 (TSEP + RIVA)^ab^	9	0	2	0
G6 (TSEP + TXT)^b^	11	0	0	0
G7 (H_3_PO_4_ + TSEP + RIVA)^b^	10	0	1	0
G8 (SU + TXT)^a^	6	2	3	0
G9 (H_3_PO_4_ + SU + TXT)^a^	3	3	5	0
G10 (H_3_PO_4_ + SU + RIVA)^b^	10	0	1	0
G11 (H_3_PO_4_ + RIVA)^b^	10	0	1	0

Values with the same superscripted letter are not significant at *p* < 0.05.

Groups 8 and 9 (SU + TXT ± H_3_PO_4_) showed samples with cohesive failure within the enamel (CF-E) and mixed failure (MF); the failure mode observed was predominantly adhesive failure (AF) in the other groups. These differences were significant at *p* < 0.05.

### Flexural modulus analysis

The flexural modulus obtained for the three bonding materials studied are summarized in Table 5.

**Table 5:** Flexural modulus obtained for each material.

Material	Flexural modulus (GPa)
TXT	10.67 ± 0.62^a^
FUJI	6.63 ± 1.99^b^
RIVA	2.42 ± 0.82^c^

Values with the same superscripted letter are not significant at *p* < 0.05.

TXT had the highest flexural modulus (10.67 GPa), followed by FUJI (6.63 GPa) and RIVA (2.42 GPa). The three flexural modulus values were significantly different at *p* < 0.05.

### Degree of conversion analysis

The DCs obtained at 1 week for the three bonding materials studied are summarized in Table 6.

**Table 6:** Degree of conversion (DC) at 1 week for each material analyzed according to the curing mode.

Bonding materials	Curing mode	DC (%)
TXT	Light-cured	70.14 ± 1.09^b^
FUJI	Light-cured	77.69 ± 2.90^a^
FUJI	Self-cured	65.08 ± 1.50^c^
RIVA	Light-cured	75.17 ± 1.90^a^
RIVA	Self-cured	61.53 ± 2.80^c^

Values with the same superscripted letter are not significant at *p* < 0.05.

The highest DC at 1 week was obtained by FUJI after light curing (77.65%), followed by RIVA after light curing (75.17%), with no significant difference (*p* > 0.05). Compared with the two previous materials, TXT post-light curing had a significantly lower DC value after 1 week (70.14%).

Without light curing, FUJI and RIVA samples had a significantly lower DC values (65.08% and 61.53%, respectively) at 1 week than before, with no significant differences between the two materials.

## Discussion

### SBS values obtained

Adhesion values for orthodontic bonding were variable and depend on a multitude of factors, such as the type of adhesive used, the design of the bracket base, the morphology of the substrate bonded to the bracket, the system of forces applied by the appliance and the technique of the practitioners [1, 2, 26].

In this study, the SBS values of metal orthodontic buttons were analyzed depending on the enamel pretreatment applied and the type of adhesive used (RMGIC or composite resin). The main objective was to determine whether Riva LC HV, which is a restorative RMGIC, provided sufficient bonding values for bonding orthodontic brackets. An additional objective was to clarify the method in which the methods of enamel pretreatment would influence SBS to optimize the bonding protocol for this material.

In our study, Group 3 (TSEP + FUJI) had the highest SBS (37.23 MPa), followed by Group 2 (OC + FUJI) (27.32 MPa). Group 6 (TSEP + TXT) obtained a significantly lower SBS value (19.38 MPa) than Groups 2 and 3. These results contradicted some studies in which SBS values were similar or higher for a primer/composite resin combination relative to FUJI [6, 27–29]. Study results differ depending on the pretreatment applied, the localization of bonding (palatal or vestibular enamel) or the addition of thermocycling to simulate intraoral aging [6, 28, 29]. For example, Ghoubril et al. [6] found similar SBS between H_3_PO_4_ + FUJI and H_3_PO_4_ + TXT groups for bonding to palatal enamel, whereas these values were relatively high for the TXT group in the case of bonding to vestibular enamel.

Polymerization of the bonding material under the bracket plays a role in its mechanical properties: the higher the DC is, the higher the SBS values [26, 30]. In orthodontics, several factors influenced polymerization quality: the type, thickness, and percentage of fillers in the bonding material, the type of light curing unit and its intensity, the distance between the light curing and the bracket, the time of light curing and the type of bracket used [26, 30]. Some authors showed better polymerization under ceramic brackets than under metal brackets because the latter were opaque to the light of the curing lamp [26, 30]; in our study, metal orthodontic buttons were chosen. Moreover, TXT was a light curing material, whereas the two RMGICs were light curing materials with intrinsic acid‒base setting reactions, which could improve their polymerization reactions [31–33]. These differences between the three materials analyzed could partly explain the higher SBS obtained by FUJI relative to TXT and RIVA.

In contrast, some scholars found higher SBS values for FUJI than for composite resins, which agreed with our results [31, 32]. Indeed, Althagafi [27] showed higher SBS values for FUJI (25.2 MPa) than for TXT (20.9 MPa), regardless of the protocol used (prior enamel etching, application of fluoride gel, bonding to healthy or eroded enamel). Authors explained their results by the improved polymerization of FUJI under the brackets [31, 32].

In our study, the SBS values of RIVA differed significantly depending on the pretreatment applied. The highest SBS value was obtained in Group 10 (H_3_PO_4_ + SU + RIVA) (25.15 MPa), whereas the lowest was obtained in Group 7 (H_3_PO_4_ + TSEP + RIVA) (17.51 MPa). This finding suggested that the use of a universal adhesive (Scotchbond Universal) could enhance the SBS of RIVA to enamel. However, the addition of TSEP, which is a conventional orthodontic self-etch primer, to the RIVA bonding protocol seemed less relevant than the addition of the universal adhesive. The results of Group 7 contradicted those of previous studies where the authors found an increase in SBS with acid etching and self-etch primer application combined with FUJI compared with the application of FUJI alone [33, 34]. Concerning the application of a universal adhesive prior to FUJI, in the field of orthodontics, no scholars have tested this combination. In restorative dentistry, authors showed a high SBS value with acid etching and universal adhesive application prior to the application of a RMGIC on composite resin or dentin [35]. Contrary to conventional self-etching adhesives, universal adhesives provided micromechanical and chemical adhesion by the interactions of functional monomers with the calcium of hydroxyapatite, which could explain the increase in the SBS values obtained [36–39].

For the groups using TXT, the application of a universal adhesive after enamel etching significantly increased the SBS values (29.30 MPa for Group 9 (H_3_PO_4_ + SU + TXT) vs. 19.38 MPa for Group 6 (TSEP + TXT) and 21.6 MPa for Group 8 (SU + TXT)). These results agreed with those of several authors who have shown an increase in the adhesion value with acid etching prior to the application of a universal adhesive but no significant difference between the application of a conventional self-etching system and a universal adhesive without prior etching [36–40]. In the case of a universal adhesive, acid etching polarized the enamel surface and exposed hydroxyl groups on its surface, helping to increase the chemical interactions of functional monomers, such as 10-MDP, with the calcium of the hydroxyapatite. This adhesive provided a stronger adhesion than a conventional adhesive [36–40].

Finally, all the groups tested in our study had bond strength values well above the minimum needed for bonding orthodontic brackets as defined by Reynolds [3] (between 5.9 and 7.8 MPa) therefore, they were clinically applicable for the three materials evaluated. Nevertheless, the adhesion values must be high enough to withstand the orthodontic forces applied during treatment but must allow debonding without enamel damage [1–3]. Thus, it is commonly accepted that the higher the SBS values are, the greater the risk of mixed or cohesive fracture within the enamel upon debonding [36–38].

### Failure modes

Our study showed samples with cohesive failure within the enamel (CF-E) and mixed failure (MF) for Groups 8 and 9 (SU + TXT ± H_3_PO_4_). These results were significantly different from the other groups tested, which had the most adhesive failure (AF). These results were confirmed by other studies. Groups 8 and 9 achieved high SBS values, and it is widely accepted that high SBS values are indicative of the quality of the bonding interface, with a correlation between a high SBS and mixed or cohesive failure within the enamel [36–38]. Cerone et al. [40] evaluated three universal adhesives and a self-etch primer for bonding orthodontic attachments and showed that there was little or no adhesive remaining on the enamel surface at debonding, showing preferential failure at the enamel/adhesive interface. This finding suggested that it was relatively easy to clean the adhesive after debonding and that there was a greater risk of enamel fracture during bracket debonding when using a universal adhesive than when using a self-etching primer. Several authors reported a greater risk of enamel fracture during debonding for composite resins than for RMGICs [28, 41, 42], for which failure occurred preferentially at the RMGIC/bracket interface or within the material with an RMGIC. There was generally additional adhesive remaining on the tooth at debonding and a reduced risk of enamel fracture [31, 32, 42]. These findings were consistent with our results where adhesive failure were observed in all RMGIC groups despite the high SBS values obtained by FUJI groups.

### Flexural modulus

In our study, the highest flexural modulus was obtained by TXT compared with FUJI and RIVA. Furthermore, FUJI obtained a higher flexural modulus than RIVA. This result was confirmed by the findings of other studies where TXT obtained good results [42–45]. In fact, composite resins had better mechanical properties and rigidity than RMGICs [5, 6, 45]. Authors explained these findings by a possible correlation between flexural modulus and SBS value: the higher the flexural modulus is, the better the SBS [43–45]. These differences in flexural modulus between the two RMGICs analyzed could partly explain the higher SBS obtained by FUJI than by RIVA.

### Degree of conversion

Our results showed the highest DC at 1 week for FUJI after light curing (77.65%), followed by RIVA after light curing (75.17%), with no significant differences (*p* > 0.05). Compared with the two previous materials, TXT after light curing had a significantly lower DC (70.14%). Without light curing, FUJI and RIVA samples had significantly lower DC values than before, with significant differences between the two materials (65.08% and 61.53%, respectively). These results were similar to those found by other authors with slightly higher DC values for RMGICs than for composite resins (~80% vs. 75% at 1 week, respectively) [46]. Furthermore, the determination of DC in a self-curing mode for the two RMGICs analyzed could indicate the presence of their intrinsic acid‒base setting reaction and a dual-cure mode combining self-curing and light curing, which is a hypothesis that was already made by several scholars [28, 29, 47]. Moreover, the higher DC value obtained for FUJI than for TXT and RIVA seemed to indicate that this material experienced improved experimental polymerization. Several scholars found that the higher the DC value is, the better its mechanical properties [46, 48–50]. These findings could explain the high SBS of FUJI obtained in our study.

### Limitations

The in vitro design of our study cannot simulate all the conditions of the oral environment to anticipate the clinical behaviors of brackets bonding with RMGIC or composite resin (presence of saliva, effects of occlusal forces and those delivered by the appliances, etc.). Otherwise, an orthodontic treatment lasts ~2 years, and the various tests, particularly for shear bond strength, are generally carried out 1 week after bonding. It might be interesting to perform these tests over prolonged periods or to use thermocycling to simulate the intraoral ageing of materials to get closer to clinical conditions. Finally, the DC measurement does not fully reflect reality because it does not consider the curing conditions of the adhesives studied under an opaque material, such as a bracket or an orthodontic button.

Clinical studies are therefore needed to validate the behaviors of the materials studied.

## Conclusions

Under the conditions of this study, the bonding of orthodontic attachments to enamel could be performed with any of the three materials. Therefore, Riva LC HV could be a competitor to Fuji Ortho or the Transbond Plus/Transbond XT combination. The use of a universal adhesive could be an effective option for optimizing the bonding protocols of these materials although there is a greater risk of enamel damage at debonding with high SBS values and cohesive fractures within the enamel observed. Clinical studies would be needed to confirm the results obtained.

## Data Availability

The data that support the findings of this study are available from the corresponding author upon reasonable request.
